# Host-Induced Gene Silencing of a *Sclerotinia sclerotiorum oxaloacetate acetylhydrolase* Using Bean Pod Mottle Virus as a Vehicle Reduces Disease on Soybean

**DOI:** 10.3389/fpls.2021.677631

**Published:** 2021-07-20

**Authors:** Megan McCaghey, Dandan Shao, Jake Kurcezewski, Ally Lindstrom, Ashish Ranjan, Steven A. Whitham, Shawn P. Conley, Brett Williams, Damon L. Smith, Mehdi Kabbage

**Affiliations:** ^1^Department of Plant Pathology, University of Wisconsin-Madison, Madison, WI, United States; ^2^Department of Plant Pathology, University of Minnesota, St. Paul, MN, United States; ^3^Department of Plant Pathology and Microbiology, Iowa State University, Ames, IA, United States; ^4^Department of Agronomy, University of Wisconsin-Madison, Madison, WI, United States; ^5^Centre for Tropical Crops and Biocommodities, Queensland University of Technology, Brisbane, QLD, Australia

**Keywords:** host induced gene silencing, RNAi, *Sclerotinia sclerotiorum*, soybean, oxalic acid, *Ssoah1*, bean pod mottle virus, VIGS

## Abstract

A lack of complete resistance in the current germplasm complicates the management of Sclerotinia stem rot (SSR) caused by *Sclerotinia sclerotiorum* in soybean. In this study, we used bean pod mottle virus (BPMV) as a vehicle to down-regulate expression of a key enzyme in the production of an important virulence factor in *S. sclerotiorum*, oxalic acid (OA). Specifically, we targeted a gene encoding oxaloacetate acetylhydrolase (*Ssoah1*), because *Ssoah1* deletion mutants are OA deficient and non-pathogenic on soybean. We first established that *S. sclerotiorum* can uptake environmental RNAs by monitoring the translocation of Cy3-labeled double-stranded and small interfering RNA (ds/siRNAs) into fungal hyphae using fluorescent confocal microscopy. This translocation led to a significant decrease in *Ssoah1* transcript levels *in vitro*. Inoculation of soybean plants with BPMV vectors targeting *Ssoah1* (pBPMV-OA) also led to decreased expression of *Ssoah1*. Importantly, pBPMV-OA inoculated plants showed enhanced resistance to *S. sclerotiorum* compared to empty-vector control plants. Our combined results provide evidence supporting the use of HIGS and exogenous applications of ds/siRNAs targeting virulence factors such as OA as viable strategies for the control of SSR in soybean and as discovery tools that can be used to identify previously unknown virulence factors.

## Introduction

*Sclerotinia sclerotiorum* is a broad host range fungal pathogen that infects many dicotyledonous plants worldwide. It is the causal agent of Sclerotinia stem rot (SSR; or white mold) on soybean, a challenging and significant yield-limiting disease (Allen et al., 2017). SSR development is heavily influenced by the weather, and disease onset is favored by cool and wet conditions during flowering (Workneh and Yang, 2000; Peltier et al., 2012; Willbur et al., 2019). SSR causes substantial yield losses to soybean globally (Wrather et al., 2010), and between 2010 and 2014 SSR resulted in yield losses between 150 million and 1.1 billion kgs in the United States, annually (Allen et al., 2017). Current SSR management strategies have limited efficacy and comprise of integrated cultural, chemical, and biological control practices (Peltier et al., 2012; Willbur et al., 2019). Disease control is complicated by the lack of complete genetic resistance to SSR, however, breeding efforts have identified partially resistant soybean genotypes in the laboratory and through field trials (Boland and Hall, 1986; Chun et al., 1987; Kim and Diers, 2000; Iquira et al., 2015; McCaghey et al., 2017).

RNA interference (RNAi) is a gene silencing process that utilizes complementary RNA particles to target mRNA and inhibit gene expression and translation. RNAi has emerged as a promising technology to control pests and pathogens within agricultural systems and has been demonstrated to reduce the aggressiveness of plant pathogenic filamentous fungi both through host-induced gene silencing (HIGS) and exogenous applications of double-stranded (dsRNA) onto the surface of plants (Yin et al., 2010; Andrade et al., 2016; Koch et al., 2016; Wang et al., 2016; Tiwari et al., 2017; Gilbert et al., 2018; Wagner et al., 2020). Spray-induced gene silencing (SIGS), involving ectopic application of dsRNAs targeting pathogen genes, has the added benefit of circumventing the genetic manipulation of plants, and thus further avoiding the regulatory expenses and public concern surrounding genetically modified organisms (GMOs). SIGS has been tested on different plant-pathosystems and was effective against various groups of plant pests and pathogens (Dubrovina and Kiselev, 2019) including viruses (Carbonell et al., 2008), insects (Li et al., 2015; Gogoi et al., 2017), and fungi (Koch et al., 2016; Wang et al., 2016; McLoughlin et al., 2018; Hu et al., 2020). Furthermore, applied dsRNAs targeting pathogen genes can be taken up and processed by plants (Koch et al., 2016; Mitter et al., 2017). For instance, spraying dsRNAs targeting a gene involved in *Fusarium graminearum* ergosterol biosynthesis on detached barley leaves inhibits fungal growth both at the application site and on distal parts of leaves (Koch et al., 2016). Due to the wide array of target options, the specificity required for RNA binding, and advances in protective materials such as clay nanosheets to adhere and protect dsRNAs on the plant surface (Mitter et al., 2017), HIGS and exogenous applications of dsRNA constitute promising crop protection technologies.

The efficacy of HIGS and SIGS approaches against fungal pathogens is dependent, in part, on the nature of the selected target sequence. Genes encoding pathogenicity determinants can constitute ideal targets of such approaches, because they are essential for pathogens to cause disease. In previous studies, silencing of pathogenicity genes by HIGS in multiple pathogens suppressed disease development (Panwar et al., 2013; Pliego et al., 2013; Yin et al., 2014, 2015; Shanmugam et al., 2017). In *S. sclerotiorum*, oxalic acid (OA) is a key factor governing its pathogenic success. OA can contribute to pathogenesis in a variety of ways, including the manipulation of the redox status of the host (Williams et al., 2011; Ranjan et al., 2018), the induction of programmed cell death (Kim et al., 2008; Kabbage et al., 2013), and the activation of degradative enzymes (Dutton and Evans, 1996). Thus, the use of HIGS to target OA production could provide a valuable tool to manage SSR and reduce fungicide use. Previous work has targeted a chitin synthase in *S. sclerotiorum* and reduced disease severity through HIGS in transgenic tobacco plants (Andrade et al., 2016). However, it is important to test this approach on specific crops of interest, identify additional useful targets that can be stacked to ensure durable resistance in a particular host, and importantly, identify and target unique sequences such as virulence factors to avoid sufficiently conserved genes across taxa to prevent off-target effects, including within beneficial fungal communities.

Herein, we target a *S. sclerotiorum oxaloacetate acetylhydrolase* (*Ssoah1*) gene that encodes an enzyme that converts oxaloacetate to oxalate and acetate. The deletion of this gene in *S. sclerotiorum* abolished OA accumulation and resulted in restricted lesions in which the infectious hyphae gradually lose viability on tomato, soybean, and *Arabidopsis* (Liang et al., 2015 and Xu et al., 2015). Additionally, *Ssoah1* expression appears to play a particularly critical role during infection of soybean, compared to other *S. sclerotiorum* hosts (Westrick et al., 2019 and Xu et al., 2015). Using a combination of confocal microscopy, gene expression studies, small RNA (sRNA) sequencing, and virus-induced gene silencing, we show that *S. sclerotiorum* mycelia take up environmental double-stranded and small interfering (ds/siRNAs) targeting *Ssoah1*, leading to the downregulation of *Ssoah1* gene expression both *in vitro* and *in planta*. *In planta*, silencing assays were conducted using bean pod mottle virus (BPMV)-mediated HIGS. Importantly, our HIGS assays significantly suppressed *S. sclerotiorum* development and disease onset. This work demonstrates that RNAi strategies provide an additional tool for improving soybean resistance to *S. sclerotiorum* and managing SSR.

## Materials and Methods

### *In vitro* Synthesis of dsRNA and siRNA

RNA was extracted from actively growing *S. sclerotiorum* mycelia using a Maxwell RSC Plant RNA Kit (Promega) and cDNA was synthesized using a ProtoScript First Strand cDNA Synthesis Kit (New England Biolabs) following manufacturers' instructions. A 365-base pair fragment corresponding to *Ssoah1* was amplified and added with a T7 promoter sequence by PCR using specific primers, T7_OA_F and T7_OA_R (Supplementary File 1). PCR reactions (50 μl) were set up using a KAPA HiFi PCR Kit (Roche Life Science) following the manufacturer's instructions and placed in a thermal cycler using the following conditions: 95°C for 3 min, 35 cycles of: 98°C for 30 s, 62°C for 15 s, and 72°C for 30 s, and last-step extension at 72°C for 30 s. The resulting PCR product was purified using a Zymoclean Gel DNA Recovery Kit (Zymo Research) and further used for *in vitro* transcription using a MEGAscript RNAi Kit (Life Technologies) according to the manufacturer's instructions. *Ssoah1*-siRNA was processed from *Ssoah1*-dsRNA using ShortCut RNase III (New England Biolabs) and subsequently purified by ethanol precipitation protocol according to the manufacturer's specifications. To perform fluorescent microscopy, *Ssoah1*-dsRNA and siRNA that were processed from dsRNA were independently labeled with Cy3 dye using a Silencer siRNA Labeling Kit (Life Technologies). Both non-labeled and labeled *Ssoah1*-dsRNA and siRNA were subsequently quantified by spectrophotometer (NanoDrop 2000, Thermo Scientific) following the protocols provided in the user guides for the MEGAscript RNAi Kit and Silencer siRNA Labeling Kit. GFP-dsRNA was used as a negative control, and a 353 bp-fragment of the GFP gene was also selected for *in vitro* transcription of GFP-dsRNA using procedures mentioned above and the primer pair, T7_GFP_F and T7-GFP-R (Supplementary File 1).

### Detection of Fluorescently Labeled ds/siRNAs in *Sclerotinia sclerotiorum*

A 5-mm hyphal plug of *S. sclerotiorum* from the leading edge of a 2-day old fungal culture was placed on a cellophane layer on the surface of a Petri plate containing potato dextrose agar (PDA). After 2 days, 1 ml of potato dextrose broth (PDB) was added to the Petri plate, and fungal mycelia were collected from cellophane and transferred into a 1.5 ml Eppendorf tube. After centrifugation at 4,000 rpm for 5 min, the supernatant was removed, and mycelia were resuspended in 200 μl of PDB. Next, 4 μg of Cy3-labeled *Ssoah1*-dsRNA/siRNA were mixed into the PDB containing *S. sclerotiorum* mycelia and incubated on a shaker at room temperature at 100 rpm for 13 h. Cy3-*Ssoah1*-dsRNA/siRNA treated *S. sclerotiorum* mycelia were washed by adding 800 μl of sterile H_2_O, and mycelia were then collected by centrifugation, at 4,000 rpm for 5 min, for microscopy. To produce protoplasts from Cy3-*Ssoah1*-dsRNA/siRNA treated fungal material, mycelia collected from cellophane on PDA were suspended in 1 ml PDB and transferred into a 25 ml flask. After 18 h of incubation with 20 μg dsRNA or siRNA, mycelia were collected by centrifugation, and protoplasts were processed by adding 2.4 ml protoplasting buffer containing 10 mg/ml lysing enzyme (modified from Rollins, 2003). To determine if *S. sclerotiorum* takes up dsRNAs non-selectively, 4 μg of Cy3-labeled siRNA with no targets in *S. sclerotiorum* (Silencer Cy3-labeled Negative Control No. 1 siRNA, Thermo Fisher Scientific) was also applied to the fungus exogenously. Water, applied to *S. sclerotiorum*, was used as negative control for microscopy. Mycelia and protoplasts were visualized by confocal microscopy using a Zeiss LSM 710.

### RNA Extraction, cDNA Synthesis, and RT-qPCR

A 5-mm *S. sclerotiorum* hyphal plug was placed into a 25-ml flask with 7 ml PDB at pH 8. Non-labeled *Ssoah1*-dsRNA were applied to PDB to make a final dsRNA concentration of 500 ng/ml, and the flasks were kept at room temperature on a shaker at 100 rpm. The application of GFP-dsRNA in PDB with *S. sclerotiorum* hyphal plugs, maintained in the same conditions as above, was considered a negative control for this experiment. Mycelia were collected at 96 h after placement in the PDB. RNA extraction and cDNA synthesis were performed as described previously. The transcriptional level of *Ssoah1* was determined by RT-qPCR using synthesized cDNA as a template. Reactions (20 μl) were set up using SsoFast EvaGreen Supermix (Bio-Rad) according to the manufacturer's instructions. Quantitative PCR (qPCR) reactions were conducted using the Bio-Rad CFX96 Real-Time System and following program: 95°C for 30 s, followed by 40 cycles of: 95°C for 5 s, and 58°C for 5 s. Melt curves were generated by heating from 65°C to 95°C with 0.5°C increments. Relative expression of *Ssoah1* to *S. sclerotiorum H3* were calculated as 2^−Δ*Ct*^ (Livak and Schmittgen, 2001) prior to calculating expression relative to the GFP negative control. Experiments were repeated three times with at least three biological replicates for each repetition. Primers used for qPCR are listed as follows: Ssoah1_F1, Ssoah1_R1, H3_F1, and H3_R1 (Supplementary File 1). In conjunction with expression assays, PDB was collected from 7 ml cultures containing 500 ng/ml of dsRNA or GFP at 24, 48, and 72 h to analyze oxalate production using the method described in Willbur et al. (2017) (Supplementary File 2). Plates were read at 590 nm using a Bio-Rad iMark (Bio-Rad Laboratories) microplate reader.

### Construction of BPMV Silencing Vectors

In order to silence the fungal gene *Ssoah1*, a 365 base pair sequence (Supplementary File 3) was selected within the mRNA of *S. sclerotiorum* strain 1980 (GenBank Accession XM_001590428). Total RNA was extracted from *S. sclerotiorum* using Trizol, and cDNA was synthesized via reverse transcription-PCR (RT-PCR) using an AMV first strand cDNA synthesis kit (New England Biolabs, Catalog # E6550). Through PCR with a high-fidelity DNA polymerase (KAPA HiFi®, Kapa Biosystems), BAMH1 restriction sites (to form “sticky ends”) were incorporated onto the double-stranded cDNA using specific primers (BAMH1_OA_F and BAMH1_OA_R, Supplementary Table 1), and the following PCR conditions: 95°C for 2 min followed by 35x at 98°C for 20 s, 68°C for 30 s, 72°C for 15 s, and a final extension at 72°C for 4 min. After digestion and gel purification (QIAquick Gel Extraction Kit®, QIAGEN), the construct was ligated into the viral vector RNA2 plasmid, pBPMV-IA-V1 to form pBPMV-OA (Zhang et al., 2010). The vector plasmids were then transformed into DH5α competent cells using 5 μl of the purified ligation product per 60 μl of competent cells, a 30 min ice incubation, 45 s heat shock in a 42°C water bath, and incubation in 500 μl of Luria broth (LB) for 1 h at 37°C. Colony PCR with MyTaq^TM^ Red Mix (Bioline) was used to confirm successfully transformed clones. After an initial step for cell breakage at 95°C for 6 min, 35 cycles (95°C for 15 s, 68°C for 15 s, and 72°C for 10 s) were performed to confirm successful transformations, and colonies from a mini preparation were sent for sequencing to confirm antisense insertion. Using glycerol stocks, midi preparations were conducted (Fast Ion Plasmid Midi Kit®, IBI Scientific) for subsequent biolistic inoculations.

### Plant Inoculation With BPMV Vectors

The modified system, used in this work and developed by Zhang et al. (2010), uses bean pod mottle virus (BPMV) which is bipartite. The two viral segments consist of a 6 kb RNA1 segment (pBPMV-IA-R1M) encoding viral proteins needed for genome replication and a 3.6 kb RNA2 segment (pBPMV-IA-V1) that encodes viral proteins needed for capsid assembly and movement and contains the cloning site where the silencing construct was inserted. As described in the protocol by Whitham et al. (2016), the RNA1 and RNA2 segments were co-bombarded into 10-day old Williams 82 (PI 518671) seedlings using gold particles coated with plasmid DNA. Two inoculations were made per plant, with one on each unifoliate leaf. Prior to inoculations, unifoliate seedlings were forced to etiolate in the dark for 24 h. After inoculations, plants were sprayed with water and kept in plastic bags for 24 h to maintain humidity. The inoculated plants were placed in a growth chamber at 22°C during the day and 20°C at night on a 16 h photoperiod. Soil was checked daily to determine if water was needed, and the plants were fertilized once weekly with Miracle-Gro® (Scotts Miracle-Gro Co.).

Successful infection of Williams 82 soybean plants was confirmed visually, as phenotypes resembled those of plants infected with BPMV, including rugosity and mottling (Supplementary File 4). Further confirmation using RT-PCR was also performed (Supplementary File 5), using the primers for silencing construct development, and one corresponding to the RNA2, VS_R2_F, and VS_R2_R (Supplementary File 1). Williams 82 soybean plants confirmed to contain the viral construct were lyophilized and stored at −80°C to be used in subsequent rub inoculations of more soybean plants. These inoculations were performed by grinding 50 mg of lyophilized tissue in 50 mM potassium phosphate buffer and rubbing the plant serum onto the soybean variety Traff (PI 470930), as described in Whitham et al. (2016). The Traff-BPMV HIGS system was previously validated by Ranjan et al. (2018). Traff has more tolerance to BPMV than Williams 82 and *Sclerotinia* and BPMV are able to co-infect in this system. Observations suggest that BPMV-infected Williams 82 inhibits *S. sclerotiorum* infection (Grau, data not shown). Viral symptoms of mottling and rugosity were apparent but milder in Traff than in Williams 82 (Supplementary File 6). RNA was extracted from the third trifoliates of plants with viral symptoms, from one experimental replicate, and plants were confirmed to contain the silencing constructs via RT-PCR and sequencing.

### Characterization of Processed sRNAs

To confirm the processing of siRNA from pBPMV-OA, a high concentration of total RNA was extracted from three leaves in three biological replicates from the pBPMV-OA and pBPMV-EV- containing plants. Leaves for RNA extractions were collected from the variety Traff, prior to inoculating soybeans symptomatic for BPMV with *S. sclerotiorum* in one experimental replicate of the expression experiment. The third trifoliate leaves were immediately frozen in liquid nitrogen. Two grams of leaf tissue per biological replicate was ground into fine powder using liquid nitrogen in a mortar and pestle and used in RNA extractions. A phenol chloroform extraction with a LiCl purification was performed as described in Aragão et al. (2013) to yield high RNA concentrations of 1.2–2.8 μg/μl. A cDNA library was constructed by Novogene (CA, USA) using a Small RNA Sample Pre Kit, and Illumina sequencing was conducted according to company workflow, using 20 million reads. Raw data were filtered for quality as determined by reads with a quality score >5, reads containing N <10%, no 5′ primer contaminants, and reads with a 3′ primer and insert tag. The 3′ primer sequence was trimmed and reads with a poly A/T/G/C were removed.

### Pathogen Aggressiveness Assays

In order to assess the aggressiveness of *S. sclerotiorum* on plants containing pBPMV-OA and plants containing pBPMV-EV constructs, BPMV-symptomatic Traff plants were inoculated using the cut petiole method as detailed by Peltier and Grau (2008), and lesions were measured 24–120 h post inoculation (HPI). Five pots, completely randomized per biological replicate and treatment, were thinned to one to three symptomatic plants per pot. Viral symptoms were apparent by the V3-V5 (vegetative stage at which the plant had three to five trifoliates) growth stage.

At the V4-V5 growth stage, the third trifoliate was excised at 2 cm and inoculated with *S. sclerotiorum* strain 1980. All cultures were grown in the same manner prior to inoculations. The isolate was obtained from sclerotia harvested from plants grown in a growth chamber, surface disinfested for 1 min in 95% ethanol and 1 min in 10% concentrated household bleach (8.25% NaClO, prior to dilution), and grown on standard, 15-mm deep Petri plates containing PDA, before sub-culturing onto 20-mm Petri plates with a thicker depth of PDA prior to inoculations. After 3 days of growth on these latter plates, a plug from the leading edge of mycelia was cut using an inverted one-ml pipette tip. The inverted pipette tip with agar plug, was then slid onto the excised petiole, which was cut to 2 cm prior to inoculation. Lesions were measured with digital calipers 24–120 HPI. These data were used to compare lesion size, across time points, and used to calculate the area under the disease progress curve transformation (Shaner and Finney, 1977). This experiment was repeated three times.

### Plant Expression Assays

Similarly, for expression assays, Traff soybean plants were challenged with *S. sclerotiorum* strain 1980 using the cut petiole technique at the third petiole when plants were at the V4 or V5 growth stage. Expression assays were conducted in experiments independent of the pathogen aggressiveness assay. However, the 120 h time point used in expression assays included plant samples from the pathogen aggressiveness assay, since lesions in the aggressiveness assay were last measured at 120 h. In order to understand whether expression decreased in plants containing the silencing construct compared to plants with pBPMV-EV, 6 cm of stem tissue (3 cm above and 3 cm below the inoculation site) was collected 48–120 HPI and immediately frozen in liquid nitrogen. One-to-three stems per pot were combined for RNA extraction and three biological replicates were used for extractions. Expression experiments were repeated three times. Tissues were ground in liquid nitrogen using a mortar and pestle, and RNA was extracted using a Maxwell® RSC Plant RNA Kit. Two primer pairs were used for RT-qPCR to determine whether expression was reduced in plants containing pBPMV-OA (as described previously for expression analyses with exogenous applications of RNA). One primer pair (Ssoah1_F1 and Ssoah1_R1) corresponded to *Ssoah1*, outside of the region used for the silencing precursor, as to not amplify sequences from the silencing vector, and another corresponded to the *S. sclerotiorum* endogenous control, *Histone H3* (H3_F1 and H3_R1). Relative expression of *Ssoah1* to *S. sclerotiorum H3* were calculated as 2^−Δ*Ct*^ (Livak and Schmittgen, 2001) prior to calculating expression relative to plants containing the empty vector negative control, pBPMV-EV.

### Statistical Analyses

Expression, AUDPC, and lesion size differences were evaluated using a mixed-model analysis of variance (ANOVA) using PROC GLIMMIX in the SAS statistical software package (v 9.4, SAS Institute, Inc. Cary, NC, United States). Significance was reported at the α = 0.05 significance level. For the lesion size differences in aggressiveness assays, a lognormal distribution and compound symmetry covariance structure were used in the model for data analyses.

## Results

### Uptake of External RNAs by *Sclerotinia sclerotiorum*

**Figure 1:**
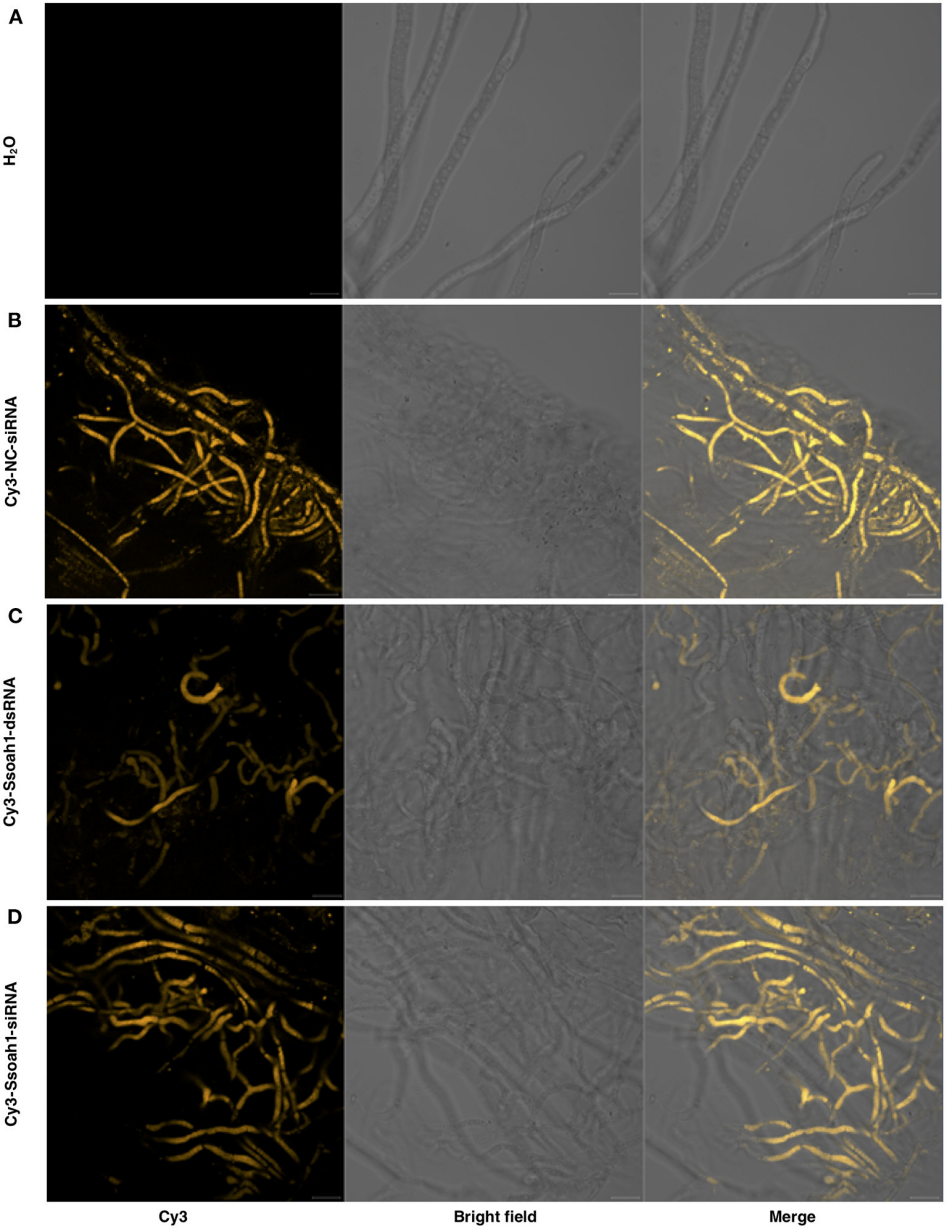
External RNAs are taken up by *Sclerotinia sclerotiorum*. Treatments were added to potato dextrose broth (PDB) containing 2 day old *S. sclerotiorum* mycelia originally grown on potato dextrose agar (PDA). Fluorescent signals were detected using confocal fluorescent microscopy. **(A)** An application of water, as a negative control, yielded no signal. **(B)** The positive control, application of Cy3-labeled-nocoding siRNA (Cy3-NC-siRNA, with no specific targets in *S. sclerotiorum)*, **(C)** 4 μg application Cy3-labeled-*Ssoah1*-dsRNA, and **(D)** application of 4 μg of Cy3-labeled-*Ssoah1*-siRNA (targeting *Ssoah1* in *S. sclerotiorum*) produced fluorescent signals that were detected 13 h post inoculation. Scale bars = 20 μm.

Effective HIGS strategies are reliant on the ability of fungal organisms to uptake external ds/siRNAs. Accordingly, we tested whether such molecules can translocate into *S. sclerotiorum* hyphae. Double-stranded RNAs (dsRNAs) corresponding to a *S. sclerotiorum Ssoah1* were transcribed *in vitro*, processed into small interfering RNAs (siRNAs) and labeled with Cy3 fluorescent dye. Fluorescent signals were detected in *S. sclerotiorum* hyphae, after a two-day growth period in potato dextrose broth (PDB) supplemented with Cy3-labeled *Ssoah1*-dsRNA/siRNAs. Clear, fluorescent signals were detected in Cy3-*Ssoah1*-dsRNA/siRNA treated samples using fluorescent confocal microscopy, but not in the negative control (Figure 1). To further confirm that RNAs were within the fungal cellular space, rather than on the surface of the hyphae, protoplasts were generated from Cy3-*Ssoah1*-dsRNA/siRNA treated mycelia. Similarly, fluorescent signals were clearly visible within protoplasts, indicating their translocation across cell walls and plasma membranes (Supplementary File 7). The uptake of environmental RNAs appears to occur readily in *S. sclerotiorum*, as Cy3-labeled siRNAs with no specific targets in this fungus were also observed in fungal mycelia and protoplasts (Figure 1 and Supplementary File 7). No fluorescence was observed in water-treated *S. sclerotiorum* mycelia or protoplasts (Figure 1 and Supplementary File 7). Taken together, these results indicate that environmental RNAs can freely translocate into *S. sclerotiorum* cells, and can thus potentially be used to target gene expression within the pathogen.

### External Application of RNAs Targeting *Ssoah1* Reduced Transcript Levels *in vitro*

**Figure 2:**
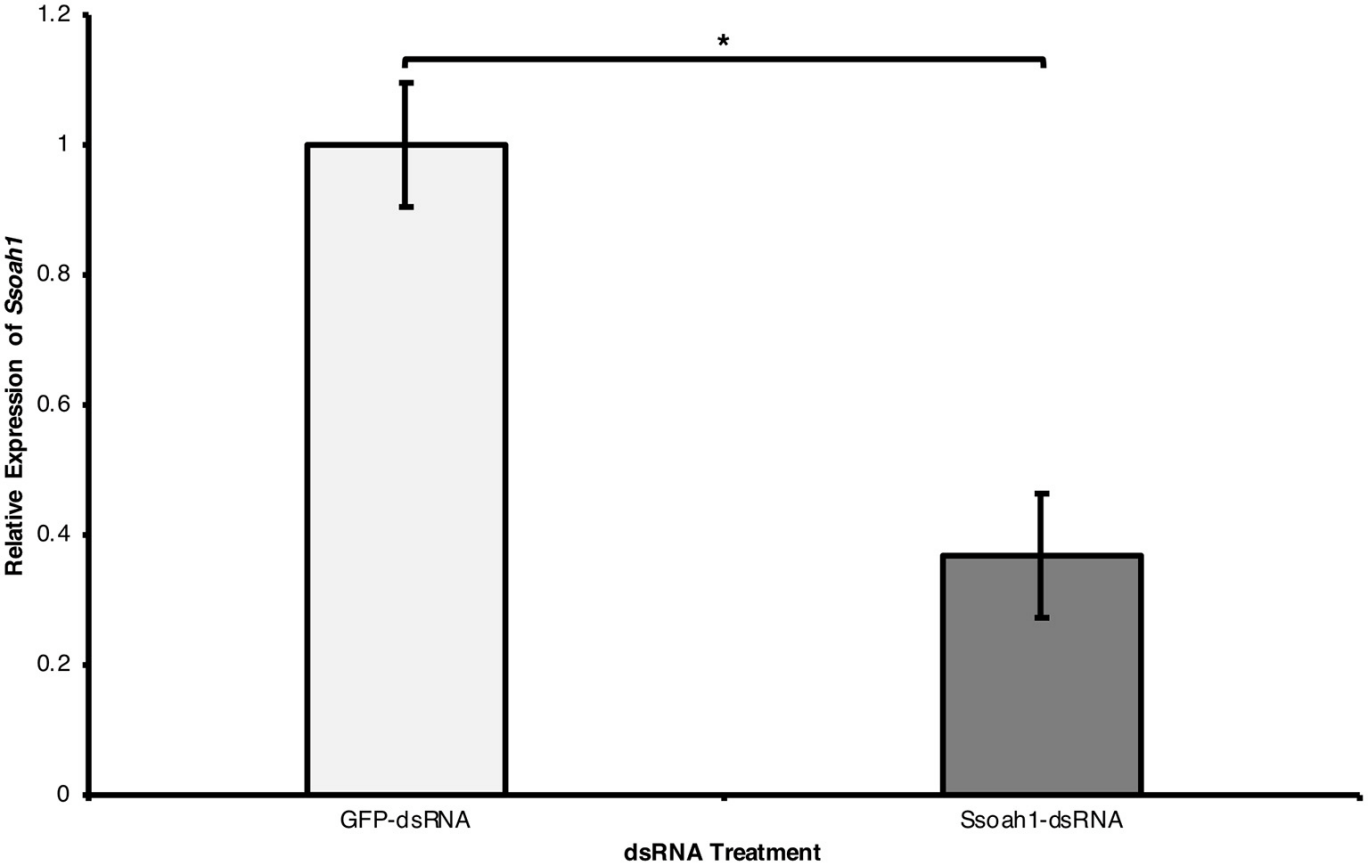
External uptake of *Ssoah1*-dsRNA decreased *Ssoah1* transcript levels in *Sclerotinia sclerotiorum*. A concentration of 500 ng/mL of *Ssoah1*-dsRNA were applied to potato dextrose broth (pH 8) and a potato dextrose agar plug containing mycelia of *S. sclerotiorum* was placed in the broth. Treatment with a same concentration of GFP-dsRNA served as negative control. Mycelia were collected 96 h after addition to PDB and the transcript level of *Ssoah1* were analyzed. Relative expression of *Ssoah1* was normalized to *S. sclerotiorum* H3 and relative to the GFP negative control. *Ssoah1* transcript levels were significantly reduced across three experimental replicates (*p < 0.01). Error bars represent ± standard errors of three (for R1 and R2) or for (for R3) biological replicates across three experimental repetitions.

We next tested whether the external application of dsRNAs leads to the silencing of the target gene. In three independent experiments, actively growing *S. sclerotiorum* hyphal plugs were placed in potato dextrose broth (PDB) containing *in vitro* transcribed *Ssoah1*-dsRNAs; GFP-dsRNA treatment served as a negative control. Transcript levels of *Ssoah1* were assessed by RT-qPCR at 96 h post-inoculation. Compared to the GFP-dsRNA controls, *Ssoah1* gene expression was markedly reduced (by >60%) in mycelia exposed to *Ssoah1*-dsRNAs (Figure 2) (*p* < 0.01), suggesting the uptake of *Ssoah1*-dsRNAs resulted in the downregulation of *Ssoah1* transcripts in *S. sclerotiorum* and thus potentially depriving the fungus from a key virulence factor during infection. As expected, the downregulation of *Ssoah1* also corresponds to a marked decrease in OA production by the fungus (Supplementary File 2).

Overall, our *in vitro* assays show that *S. sclerotiorum* uptakes environmental RNAs targeting *Ssoah1*, and this uptake leads to reduced expression of the target gene and OA synthesis. Accordingly, we reasoned that dsRNA/siRNAs generated as part of a HIGS strategy targeting a vital virulence factor might hinder *S. sclerotiorum* pathogenic development in soybean.

### Accumulation of siRNAs Corresponding to *Ssoah1* in Soybean Mediated by BPMV

**Figure 3:**
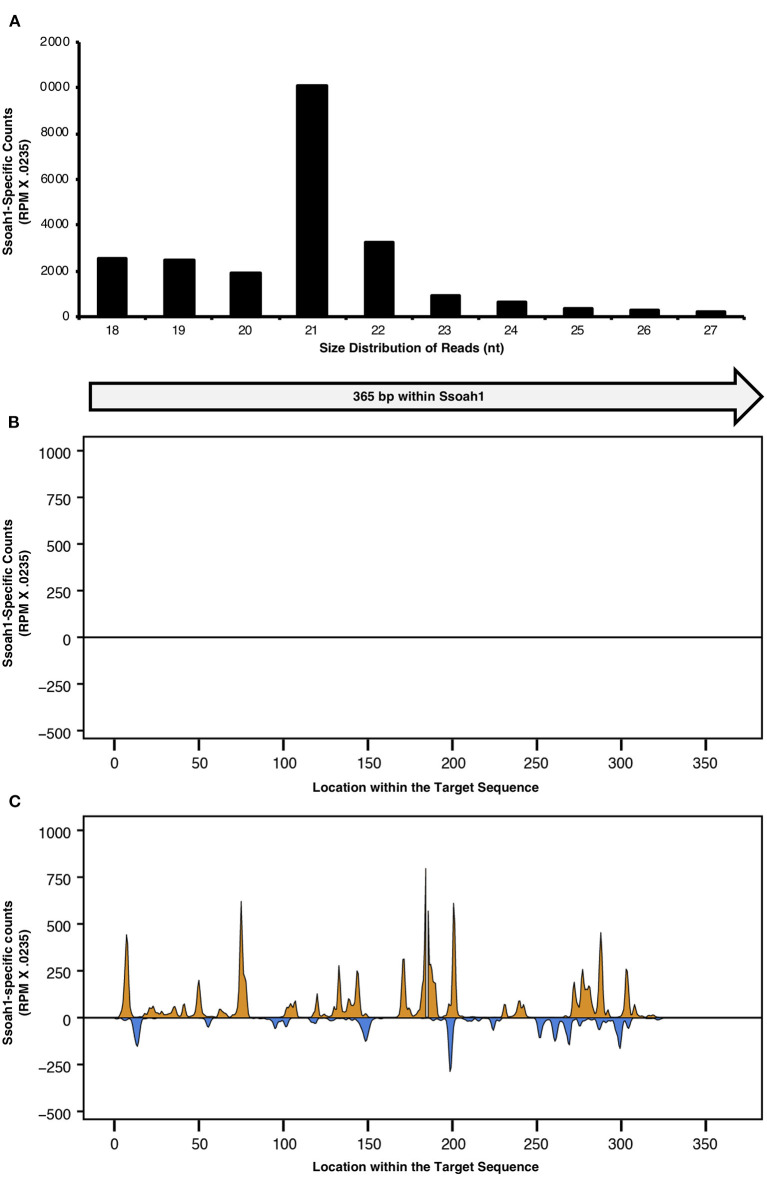
Total sRNAs were profiled from Traff soybean plants bombarded with pBPMV -OA and pBPMV-EV. The size distribution of sRNA reads <27 nt and corresponding to the 365 nt target within *Ssoah1* were normalized to a count, using the conversion factor of 0.0235 X reads per million (RPM). **(A)** The size distribution of Ssoah1-dsRNA-derived sRNAs corresponding to *Ssoah1* from a pBPMV-OA single plant are presented for sRNAs ranging from 18 to 27 nt. The 5′ end of 18–27 nt sRNAs from plants containing **(B)** pBPMV-OA and **(C)** pBPMV-EV vectors were aligned to the 365 nt sequence cDNA sequence used for silencing construct development. Antisense reads are presented in orange and sense reads are presented in blue.

With evidence that *S. sclerotiorum* could uptake exogenous siRNAs, we evaluated whether plants could express silencing constructs for uptake by *S. sclerotiorum* through HIGS. To express siRNAs targeting *Ssoah1*, a 365 bp *Ssoah1* fragment was cloned into the viral vector pBPMV-IA-V1 (Zhang et al., 2010) after the viral translation stop codon to create pBPMV-OA. Soybeans were inoculated with pBPMV-OA and an empty vector control (pBPMV-EV, negative control) using a combination of particle bombardment and rub inoculation. Small RNAs (sRNAs), sequenced from plant total RNA, were mapped to the 365 bp region carried by the pBPMV viral vector (Figure 3). Of the sRNAs profiled, 21 nt sequences were particularly abundant, with over 10,000 copies (reads per millions X.0235) estimated to be contained in leaf extracts tested (Figure 3A). This size corresponds to the 20–25 nt size characteristic of siRNAs, knowing that 21 nt siRNAs are the most efficient at mRNA degradation (Elbashir et al., 2001). These sRNAs mapped to various regions across the 365 bp cDNA silencing construct (Figures 3B,C and Supplementary File 3). The location distribution was similar in all three biological replicates from which RNA was sequenced (Figure 3 and Supplementary Files 8, 9). As expected, reads corresponding to the silencing target were only present in the plants containing the pBPMV-OA vector, and not in plants containing the empty vector, except for five, negligible reads in one of the three samples sequenced for sRNAs. Therefore, HIGS plants were confirmed to generate abundant siRNAs corresponding to the *S. sclerotiorum* gene, *Ssoah1*.

### Targeted Silencing of *Ssoah1 in planta* Using BPMV-Mediated HIGS

**Figure 4:**
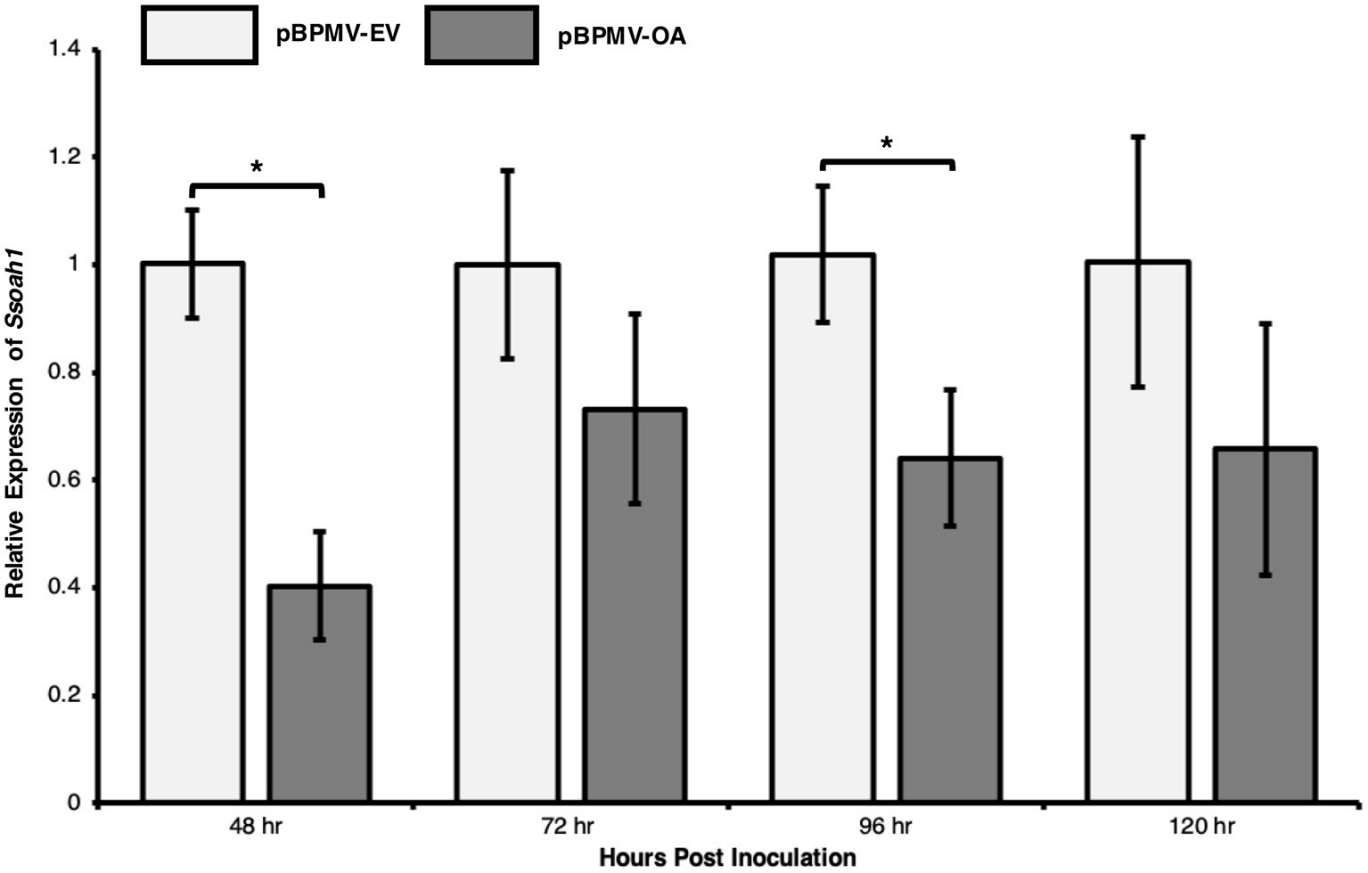
Transcript levels of the target gene, *Ssoah1*, were decreased in expression assays. Plants containing virus-induced gene silencing constructs, pBPMV-OA and empty vectors, pBPMV-EV were challenge with *S. sclerotiorum* strain 1980, using a cut petiole technique. RNA was extracted from stem tissue from three biological replicates and three experimental repetitions. Relative expression of *Ssoah1* was normalized to *S. sclerotiorum* H3 and relative to the pBPMV-EV. Expression in pBPMV-OA treated plants tended to decrease for all time points, however, the difference was significant at 48 h (*p < 0.01) and 96 h (*p* = 0.02).

We tested whether expression of the target gene, *Ssoah1*, could be reduced *in planta* using a modified BPMV vector. Plant containing pBPMV-OA and empty vector controls were challenged with *S. sclerotiorum* strain 1980 (ATCC18683) in a time-course experiment. Stem tissues were collected at 48, 72, 96, and 120 HPI for RNA extractions and subsequent RT-qPCR. Across three independent experimental repetitions, the expression of *Ssoah1* was significantly reduced in plants inoculated with the pBPMV-OA silencing vector compared to the empty vector control (Figure 4). The most significant reduction in expression was observed at 48 HPI (60% reduction, *p* < 0.01) and 96 HPI (42% reduction, *p* = 0.02; Figure 4) and all-time points trended toward a reduction in *Ssoah1* expression. Although differences were not statistically significant at 72 and 120 HPI. These results suggest that BPMV-mediated HIGS targeting *Ssoah1*, effectively reduced the expression of this *S. sclerotiorum* gene in our time course.

### Disease Development Was Impeded in BPMV-Mediated HIGS Plants

**Figure 5:**
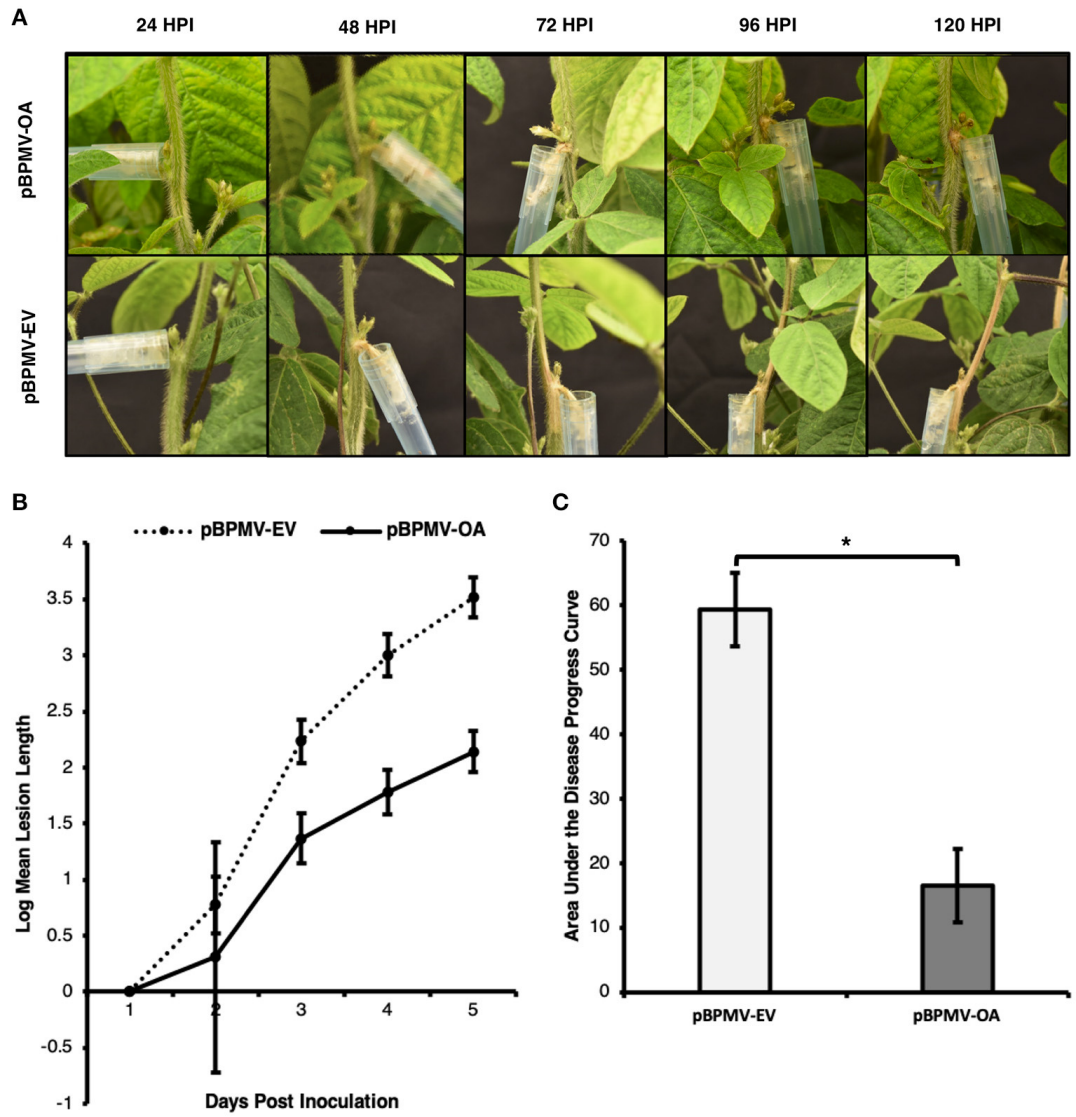
Pathogen aggressiveness was reduced in virus induced gene silencing (VIGS) assays. Plants containing VIGS silencing constructs (pBPMV-OA) and empty vectors (pBPMV-EV) were challenged with *S. sclerotiorum* strain 1980, using a cut petiole technique. Lesions were measured 1–5 days post inoculation (DPI). Data are the result of five biological replicates and three experimental repetitions. **(A)** Lesion development was delayed, and lesions were smaller in plants containing pBPMV-OA, while plants containing pBPMV-EV often showed girdling lesions at 96 h post inoculation (HPI). **(B)** Lesion size was reduced after two DPI, with an overall reduced lesion size (*p < 0.01). **(C)** The overall area under the disease progress curve was lower in plants containing pBPMV-OA (*p < 0.01).

To test whether the reduced expression of *Ssoah1* resulted in reduced pathogen aggressiveness, pBPMV-OA was introduced into the soybean variety, Traff. Symptomatic Traff seedlings were similarly challenged with *S. sclerotiorum* strain 1980 (ATCC18683) using a cut petiole method (Peltier and Grau, 2008; Ranjan et al., 2018), and lesions were measured 24-120 HPI. Visual differences in lesions were apparent, with plants containing pBPMV-EV having large, girdling lesions 96 HPI compared to markedly restricted lesions in pPBMV-OA plants (Figure 5A). Significant differences in lesion size were detected at 72 HPI and onward (Figure 5B, *p* < 0.01). Additionally, the area under the disease progress curve (AUDPC) in plants containing the silencing construct was reduced by 73% compared to pBPMV-EV plants (Figure 5C, *p* < 0.01), indicating a delay and reduction in SSR disease development. Reduced pathogen development using BPMV-mediated HIGS in soybean challenged with *S. sclerotiorum* suggest that such a strategy is effective in controlling SSR.

## Discussion

The first application of *in planta* HIGS as a disease control strategy was patented in 2008 for control of pathogenic fungi and oomycetes in *Glycine max* and *Arabidopsis* (Roberts et al., 2008). Since, various strides have been made in the application of HIGS (Peltier and Grau, 2008; Nowara et al., 2010; Tinoco et al., 2010; Yin et al., 2010; Panwar et al., 2013; Ghag et al., 2014; Andrade et al., 2016; Koch et al., 2016; Dou et al., 2020; Mahto et al., 2020) and SIGS (Wang et al., 2016; McLoughlin et al., 2018; Koch et al., 2019) in the control of a range of phytopathogens. The aforementioned works demonstrate that RNAi can be an effective gene silencing strategy in many plant-fungal interactions. Extending such methods to other fungal pathosystems could constitute an exciting alternative to chemical management, particularly in crops where breeding efforts have failed to produce highly resistant commercial cultivars. This study targets an essential pathogenicity factor of *S. sclerotiorum*, oxalic acid (OA) to enhance the resistance of soybean to Sclerotinia stem rot (SSR). Oxalic acid is an important virulence factor in *S. sclerotiorum*, where OA deficient mutants show reduced colonization and disease development (Liang et al., 2015; Xu et al., 2015). Accordingly, we targeted *Ssoah1*, a gene encoding an oxaloacetate acetylhydrolase enzyme that catalyzes the last step in OA biosynthesis, and the deletion of which abolishes OA production (Liang et al., 2015; Xu et al., 2015). Several lines of evidence presented within this study are consistent with the following conclusions: (i) environmental dsRNAs/siRNAs translocate into *S. sclerotiorum* hyphae; (ii) transcripts encoding *Ssoah1* are markedly reduced when *S. sclerotiorum* is exposed to the corresponding dsRNAs in *vitro* and *in planta*; and (iii) RNAi of *Ssoah1* using HIGS can reduce pathogen aggressiveness and SSR disease development.

We first determined whether *S. sclerotiorum* can uptake environmental RNAs, as uptake of dsRNA is not a ubiquitous feature of fungi. While exogenous dsRNA uptake occurs in the closely related *Botrytis cinerea*, recent findings indicate that the wheat pathogen *Zymoseptoria tritici* does not uptake environmental dsRNA despite encoding key components of the RNAi pathway (Wang et al., 2016; Kettles et al., 2019). After applying fluorescently labeled dsRNA/siRNA to fungal cultures, we detected fluorescent signals within fungal hyphae treated with both *Ssoah1* derived-dsRNA/siRNAs and non-targeting siRNA (Figure 1), indicating that *S. sclerotiorum* readily uptakes these molecules in a non-specific manner.

While the mechanism by which RNA molecules are able to translocate across fungal cell walls and membranes is yet to be determined, it is clear that the non-selective movement of these molecules occurs in the predominantly necrotrophic pathogen *S. sclerotiorum*. Within fungi, these translocated dsRNAs are processed to siRNA after uptake and both dsRNA and siRNA efficiently silence gene expression (Dang et al., 2011 and Koch et al., 2016). Though exceptions have been noted, ds/siRNA uptake is likely a common occurrence in the fungal kingdom considering the successful utilization of HIGS against several fungal species (Nowara et al., 2010; Tinoco et al., 2010; Yin et al., 2010; Panwar et al., 2013; Pliego et al., 2013; Ghag et al., 2014; Andrade et al., 2016; Koch et al., 2016; Cooper and Campbell, 2017; Dou et al., 2020; Hu et al., 2020; Mahto et al., 2020). Thus, RNA spray regimes or HIGS has the potential to be utilized across many fungal pathogens including *S. sclerotiorum*. Interestingly, these fungal targeted RNAs also translocate, and are amplified within plant cells as well. Long dsRNA and dsRNA-derived siRNA efficiently translocate in barley (Koch et al., 2016). Song et al. (2018) also reported the successful *in planta* amplification of siRNA targeting *Fusarium* in wheat through plant RdRp, providing evidence for the potential of sustained dsRNA/siRNA-based SIGS in a field setting. This may also alleviate concerns about the stability of such molecules in nature and may confer prolonged protection under field conditions. Spray-induced gene-silencing studies are needed to evaluate the effectiveness of exogenously-applied dsRNAs for silencing *Ssoah1* at local and distal locations, relative to the application site, in *S. sclerotiorum* hosts and to evaluate the effectiveness of SIGS targeting *Ssoah1* to manage disease in a field setting.

To confirm that these dsRNA/siRNAs were not only absorbed but actively utilized in gene silencing, *Ssoah1* transcripts in *S. sclerotiorum* were analyzed following treatment with corresponding dsRNAs *in vitro*. Oxalic acid production in *S. sclerotiorum* is a pH-responsive process and *Ssoah1* transcripts are concordantly induced in alkaline conditions (Rollins, 2003; Kim et al., 2008). To ensure that *Ssoah1* was highly expressed and a reduction of transcripts in *S. sclerotiorum* would be observable after taking up *Ssoah1*-dsRNA, the treated liquid media was alkalized to a pH of 8. As anticipated, uptake of *Ssoah1*-dsRNA did occur and resulted in significant reduction of *Ssoah1* mRNA level at 96 h after dsRNA treatment (Figure 2). These results were encouraging, and clearly show that the translocation of environmental RNAs in *S. sclerotiorum* significantly affect transcript abundance of corresponding sequences.

*In planta* assays were performed to evaluate the use of HIGS targeting *Ssoah1* to reduce the aggressiveness of *S. sclerotiorum*. We took advantage of our working virus-based HIGS system (Zhang et al., 2010; Ranjan et al., 2018) that uses BPMV as a vehicle to produce dsRNAs corresponding to our fungal target. Soybeans were inoculated with a BPMV vector containing an *Ssoah1* fragment (pBPMV-OA), and the presence of corresponding sRNAs was determined via RNA sequencing. Indeed, sRNAs aligning to *Ssoah1* were found within the expected range of siRNAs (21-24 nt, Figure 3A) in pBPMV-OA inoculated plants indicating successful processing by the soybean RNAi machinery. The sRNAs corresponding to *Ssoah1* were not detected in the empty vector (pBPMV-EV) control plants. Importantly, a marked reduction in disease symptoms and reduced AUDPC was observed in pBPMV-OA soybeans compared to control empty vector plants (Figure 5). Small lesions did develop on plants expressing *Ssoah1*-dsRNAs, but in most replicates, these lesions were not girdling the stems at 96 HPI, as occurred in plants inoculated with pBPMV-EV. This reduction in symptom development was accompanied by reduced transcript levels of *Ssoah1*, indicating that HIGS is an effective strategy for reducing expression of *S. sclerotiorum* genes in soybean and conferring resistance to this pathogen. Future work will focus on the generation and evaluation of stable, transgenic soybean plants that use HIGS to target virulence factors, such as *Ssoah1*, in *S. sclerotiorum*.

A similar, previous approach, aimed to reduce the aggressiveness of *S. sclerotiorum* by targeting the structural gene chitin synthase in tobacco (Andrade et al., 2016). This study, however, is the first case of HIGS being used to target a pathogenicity rather than developmental factors of *S. sclerotiorum*. Because *Ssoah1* orthologs are primarily present in other plant pathogenic fungi, the risk of off-target effects from this silencing construct on beneficial and non-pathogenic fungi is low. Targeting pathogenicity-related genes with RNAi in cereal crops has provided effective disease resistance to *Puccinia graminis* and the targeting of *Ssoah1* and other *S. sclerotiorum* genes may provide similar resistance in soybean (Panwar et al., 2018). Ongoing research efforts in our lab aim to identify *S. sclerotiorum* specific virulence determinants that can be utilized in this manner. This is particularly important, since targeting structural components or housekeeping genes did not yield positive results (data not shown).

Our findings pave the way for RNAi-based approaches that target virulence factors to achieve enhanced resistance to *S. sclerotiorum* in soybean and potentially other valuable field crops. Virus-based HIGS using pBPMV, as developed by Zhang et al. (2010), can also be deployed in functional studies to both understand the role of *S. sclerotiorum* genes in infecting soybean hosts and to evaluate host factors involved in *S. sclerotiorum* pathogenic development (Ranjan et al., 2018). As a novel tool in SSR management, RNAi has the potential to reduce the use of chemical control in soybean systems and to close the gap of incomplete resistance observed in commercial and public soybean cultivars (Kim and Diers, 2000; Hoffman et al., 2002; McCaghey et al., 2017; Conley et al., 2018; Huzar-Novakowiski and Dorrance, 2018).

## Data Availability Statement

Illumina sequencing data of small RNA from plants containing empty vectors and silencing vectors are publicly available through NCBI as accession GSE180131, https://www.ncbi.nlm.nih.gov/geo/query/acc.cgi?acc=GSE180131.

## Author Contributions

MM, DSh, SC, DSm, and MK designed all the experiments. MM and AR identified the silencing target. MM designed the *in planta* and exogenously applied silencing constructs and related primers. MM, JK, and AL generated pBPMV-OA silencing constructs, conducted the *in planta* assays, and analyzed data with assistance from DSm. DSh synthesized the exogenously applied dsRNA, conducted the *in vitro* experiments, and conducted microscopy and imaging. SW shared pBPMV vectors. MM, DSh, BW, DSm, and MK wrote the manuscript. All authors contributed to the article and approved the submitted version.

## Conflict of Interest

The authors declare that the research was conducted in the absence of any commercial or financial relationships that could be construed as a potential conflict of interest.
