# Supplementary material for: Host-Induced Gene Silencing of a Sclerotinia sclerotiorum oxaloacetate acetylhydrolase Using Bean Pod Mottle Virus as a Vehicle Reduces Disease on Soybean
Source: Front Plant Sci. 2021 Jul 20;12:677631. doi: 10.3389/fpls.2021.677631 (PMC8329588; doi:10.3389/fpls.2021.677631)
Supplement: Supplementary file 1 [file Presentation_1.PPTX]

## Slide 1
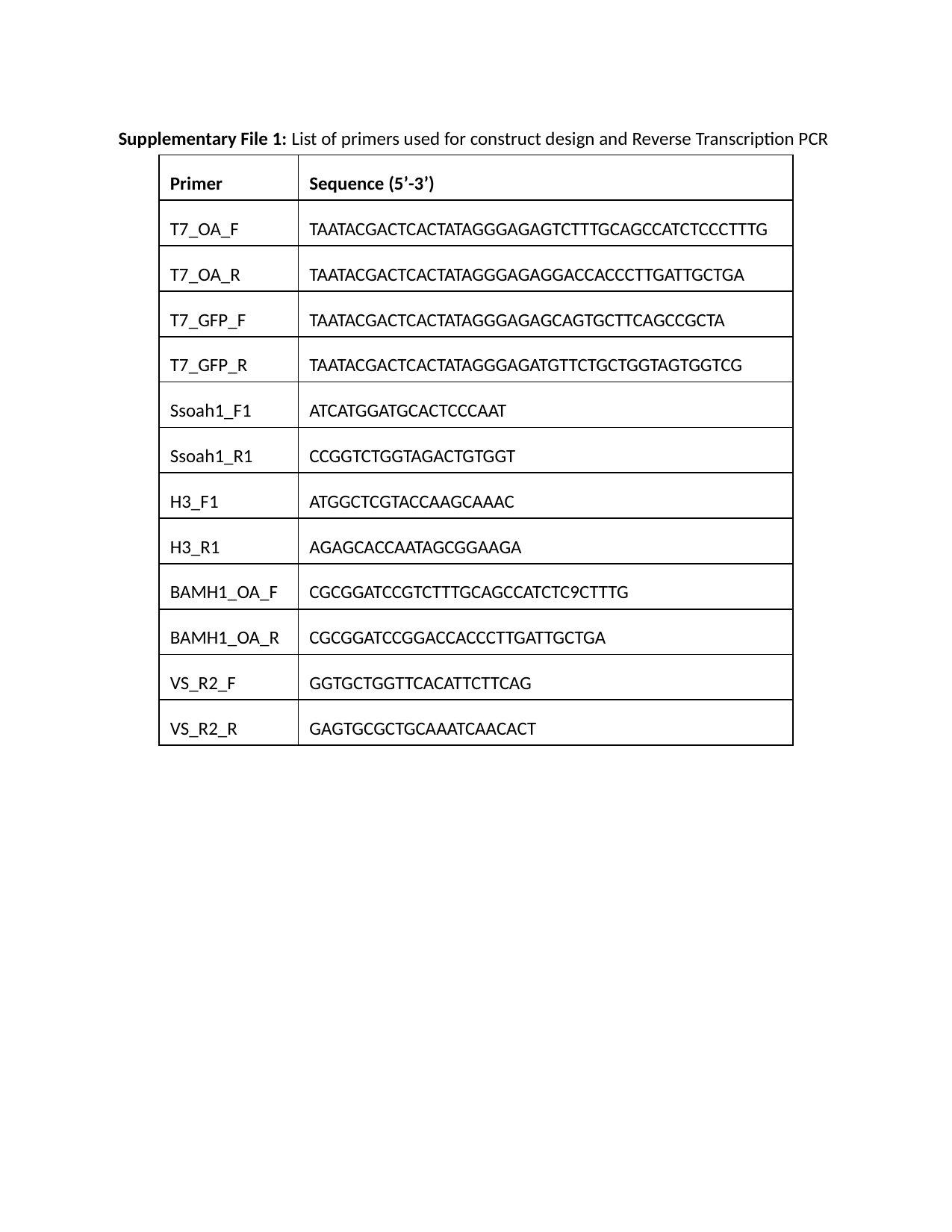

Supplementary File 1: List of primers used for construct design and Reverse Transcription PCR
| Primer | Sequence (5’-3’) |
| --- | --- |
| T7\_OA\_F | TAATACGACTCACTATAGGGAGAGTCTTTGCAGCCATCTCCCTTTG |
| T7\_OA\_R | TAATACGACTCACTATAGGGAGAGGACCACCCTTGATTGCTGA |
| T7\_GFP\_F | TAATACGACTCACTATAGGGAGAGCAGTGCTTCAGCCGCTA |
| T7\_GFP\_R | TAATACGACTCACTATAGGGAGATGTTCTGCTGGTAGTGGTCG |
| Ssoah1\_F1 | ATCATGGATGCACTCCCAAT |
| Ssoah1\_R1 | CCGGTCTGGTAGACTGTGGT |
| H3\_F1 | ATGGCTCGTACCAAGCAAAC |
| H3\_R1 | AGAGCACCAATAGCGGAAGA |
| BAMH1\_OA\_F | CGCGGATCCGTCTTTGCAGCCATCTC9CTTTG |
| BAMH1\_OA\_R | CGCGGATCCGGACCACCCTTGATTGCTGA |
| VS\_R2\_F | GGTGCTGGTTCACATTCTTCAG |
| VS\_R2\_R | GAGTGCGCTGCAAATCAACACT |

## Slide 2
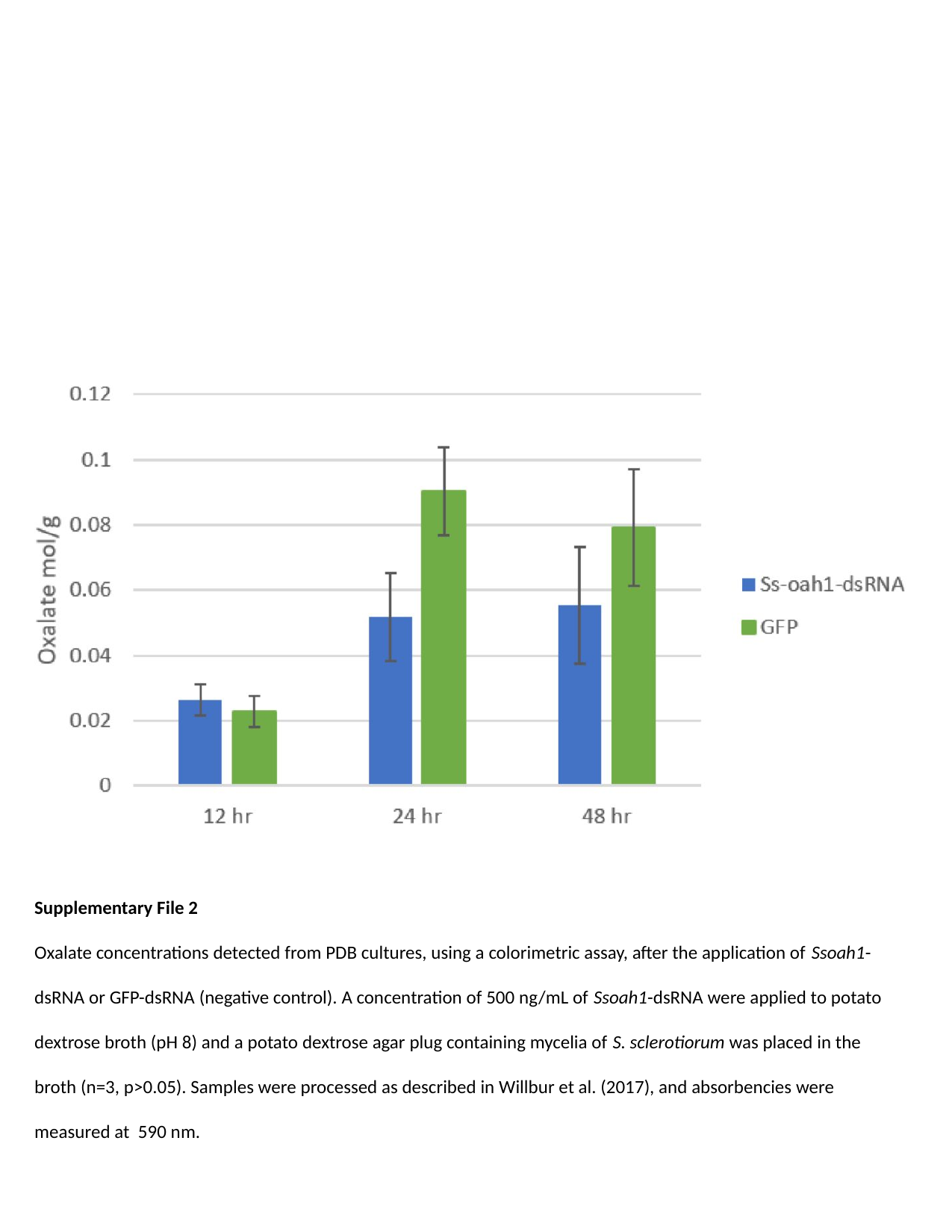

Supplementary File 2
Oxalate concentrations detected from PDB cultures, using a colorimetric assay, after the application of Ssoah1-dsRNA or GFP-dsRNA (negative control). A concentration of 500 ng/mL of Ssoah1-dsRNA were applied to potato dextrose broth (pH 8) and a potato dextrose agar plug containing mycelia of S. sclerotiorum was placed in the broth (n=3, p>0.05). Samples were processed as described in Willbur et al. (2017), and absorbencies were measured at 590 nm.

## Slide 3
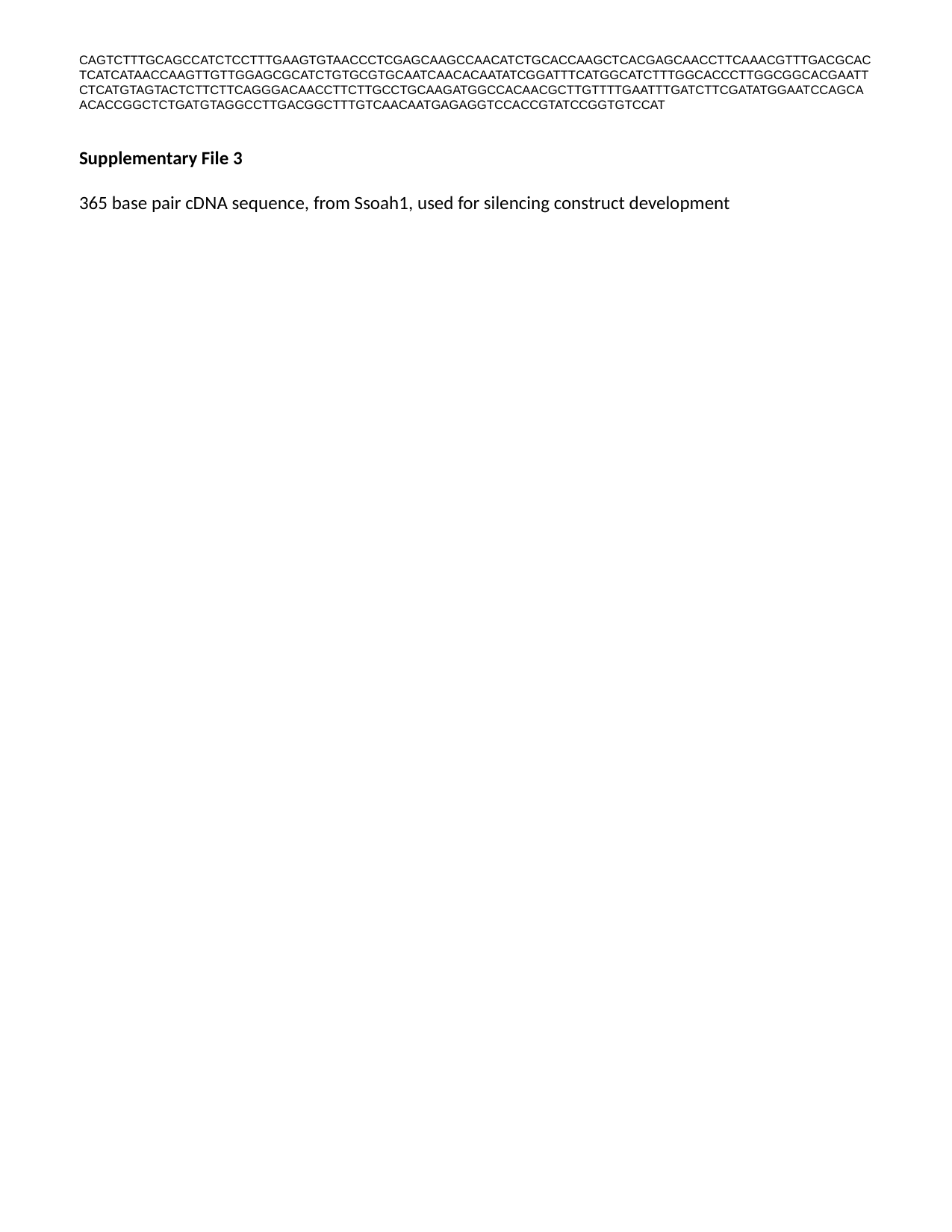

CAGTCTTTGCAGCCATCTCCTTTGAAGTGTAACCCTCGAGCAAGCCAACATCTGCACCAAGCTCACGAGCAACCTTCAAACGTTTGACGCACTCATCATAACCAAGTTGTTGGAGCGCATCTGTGCGTGCAATCAACACAATATCGGATTTCATGGCATCTTTGGCACCCTTGGCGGCACGAATTCTCATGTAGTACTCTTCTTCAGGGACAACCTTCTTGCCTGCAAGATGGCCACAACGCTTGTTTTGAATTTGATCTTCGATATGGAATCCAGCAACACCGGCTCTGATGTAGGCCTTGACGGCTTTGTCAACAATGAGAGGTCCACCGTATCCGGTGTCCAT
Supplementary File 3
365 base pair cDNA sequence, from Ssoah1, used for silencing construct development

## Slide 4
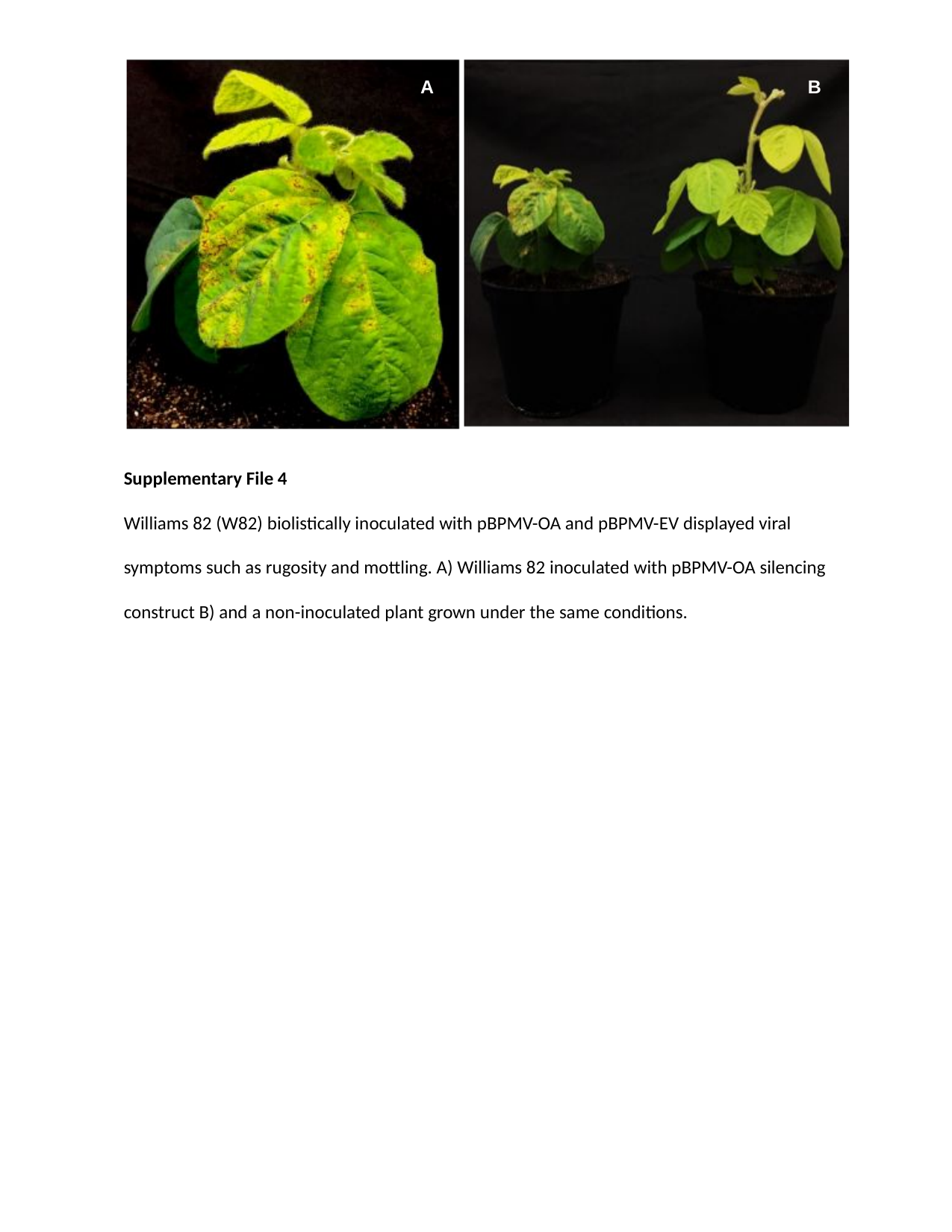

A
B
Supplementary File 4
Williams 82 (W82) biolistically inoculated with pBPMV-OA and pBPMV-EV displayed viral symptoms such as rugosity and mottling. A) Williams 82 inoculated with pBPMV-OA silencing construct B) and a non-inoculated plant grown under the same conditions.

## Slide 5
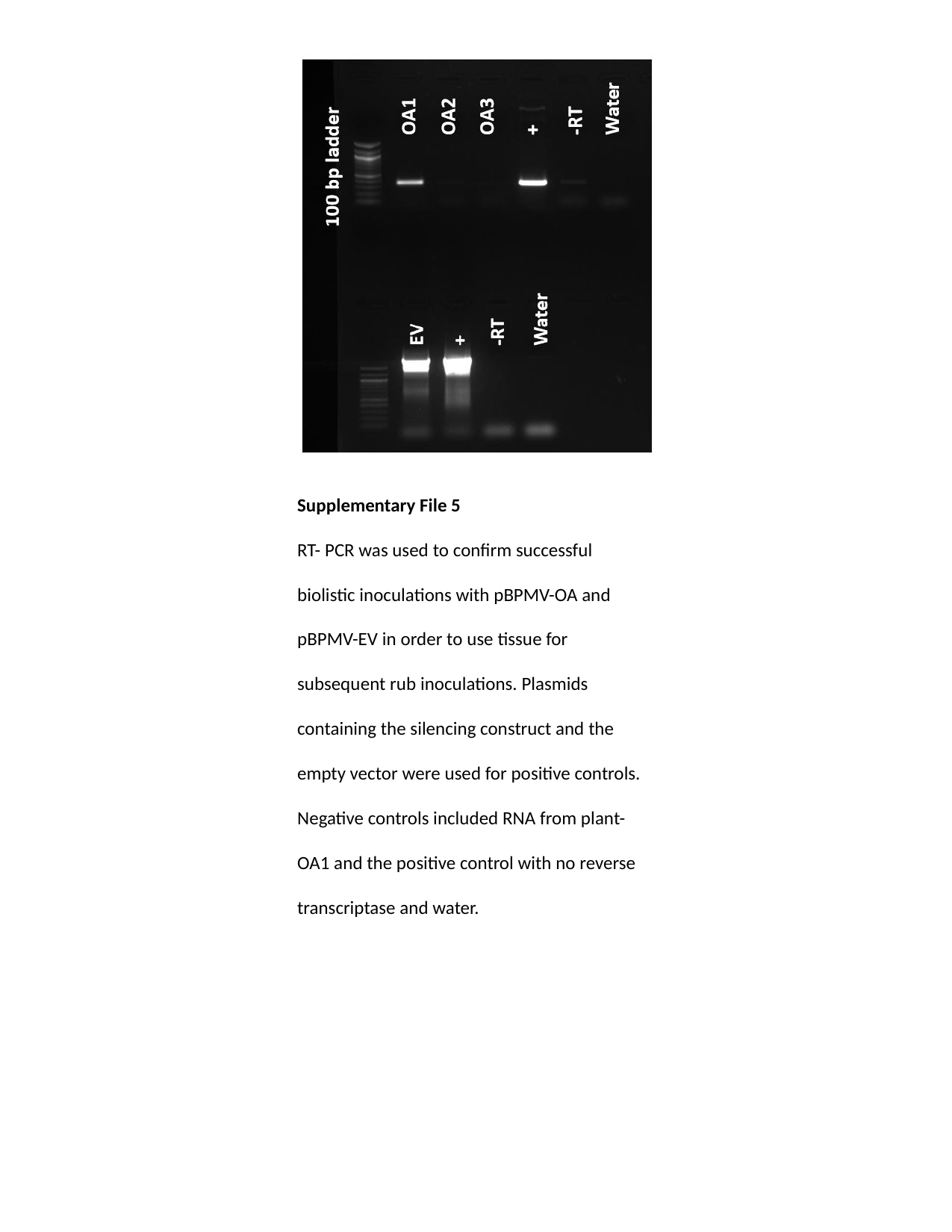

Supplementary File 5
RT- PCR was used to confirm successful biolistic inoculations with pBPMV-OA and pBPMV-EV in order to use tissue for subsequent rub inoculations. Plasmids containing the silencing construct and the empty vector were used for positive controls. Negative controls included RNA from plant-OA1 and the positive control with no reverse transcriptase and water.

## Slide 6
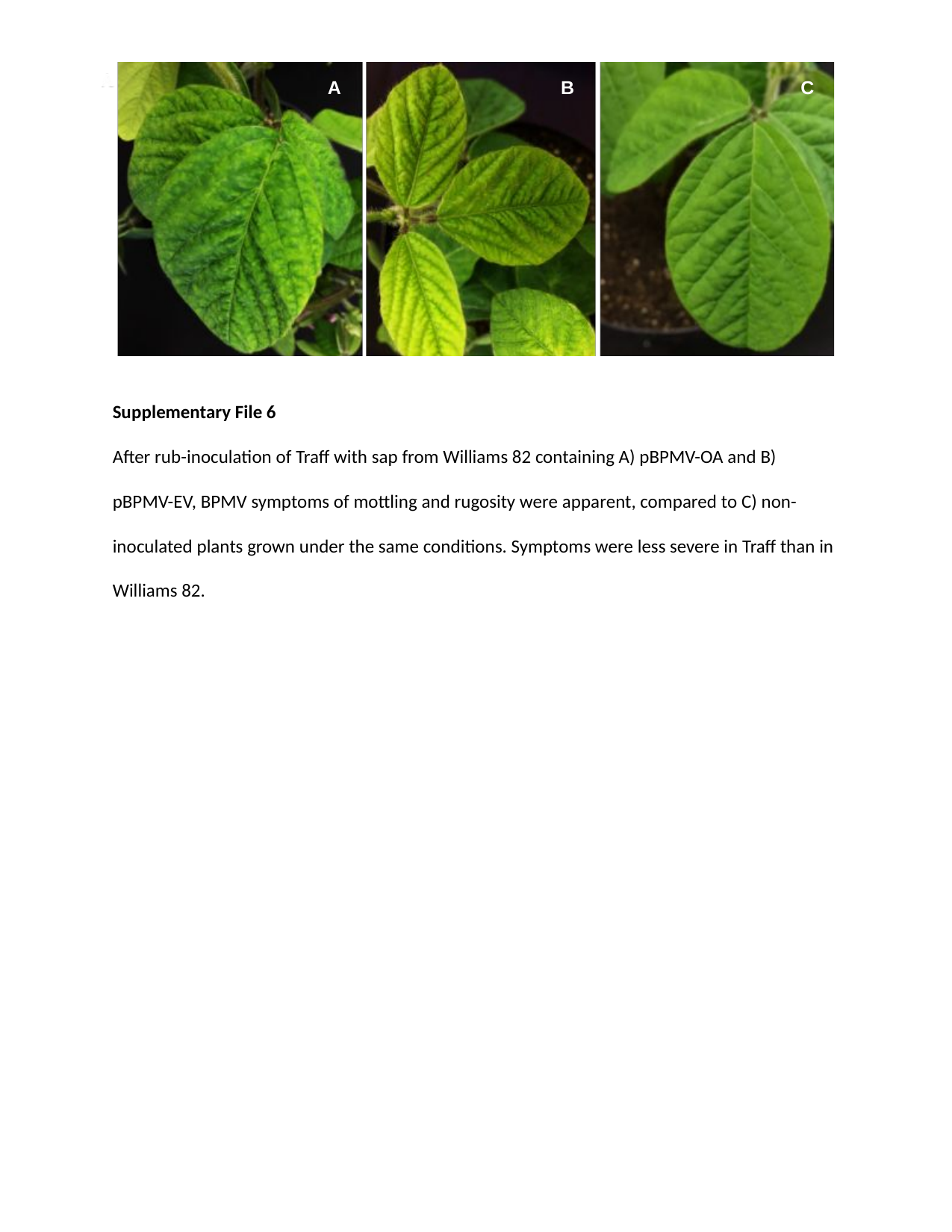

A
B
C
Supplementary File 6
After rub-inoculation of Traff with sap from Williams 82 containing A) pBPMV-OA and B) pBPMV-EV, BPMV symptoms of mottling and rugosity were apparent, compared to C) non-inoculated plants grown under the same conditions. Symptoms were less severe in Traff than in Williams 82.

## Slide 7
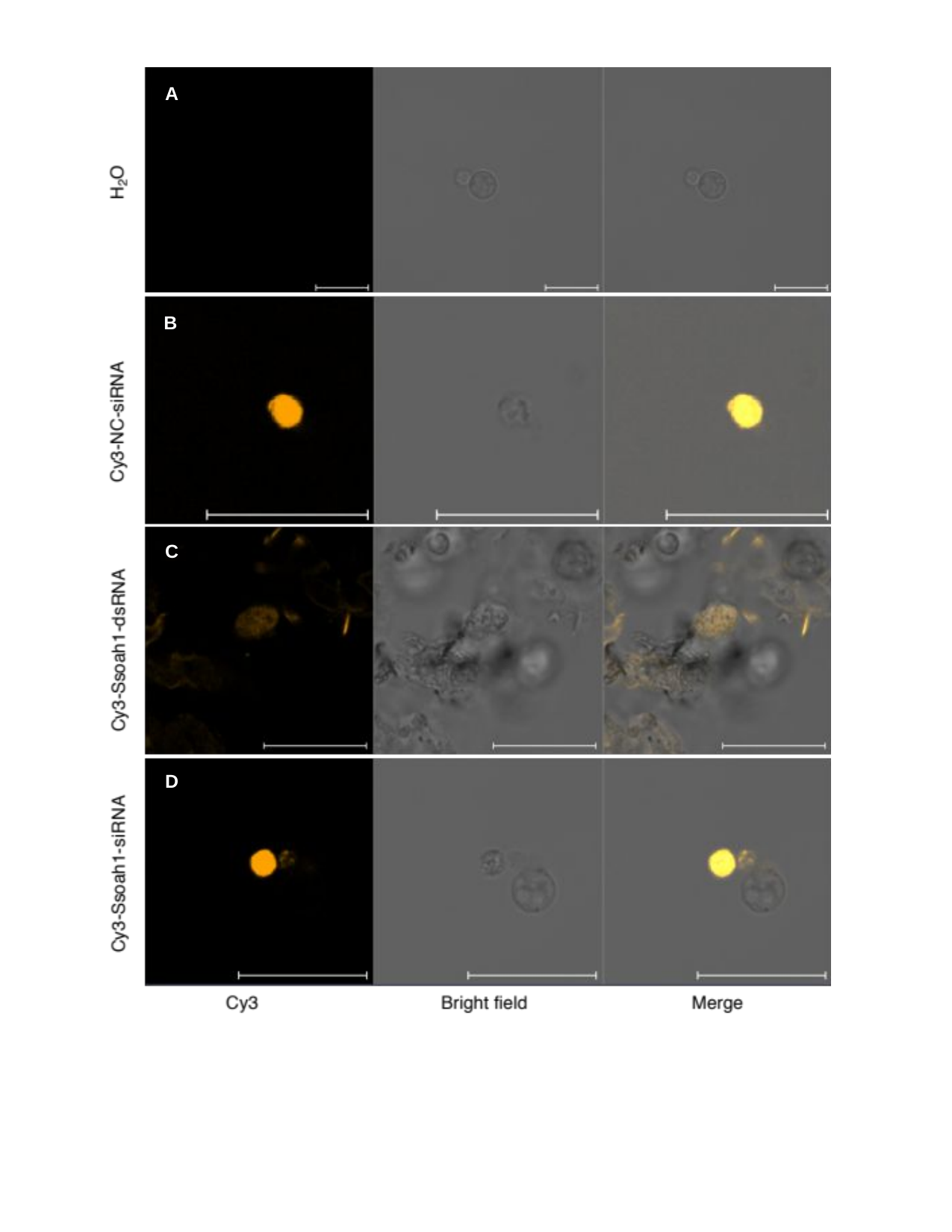

A
B
C
D

## Slide 8
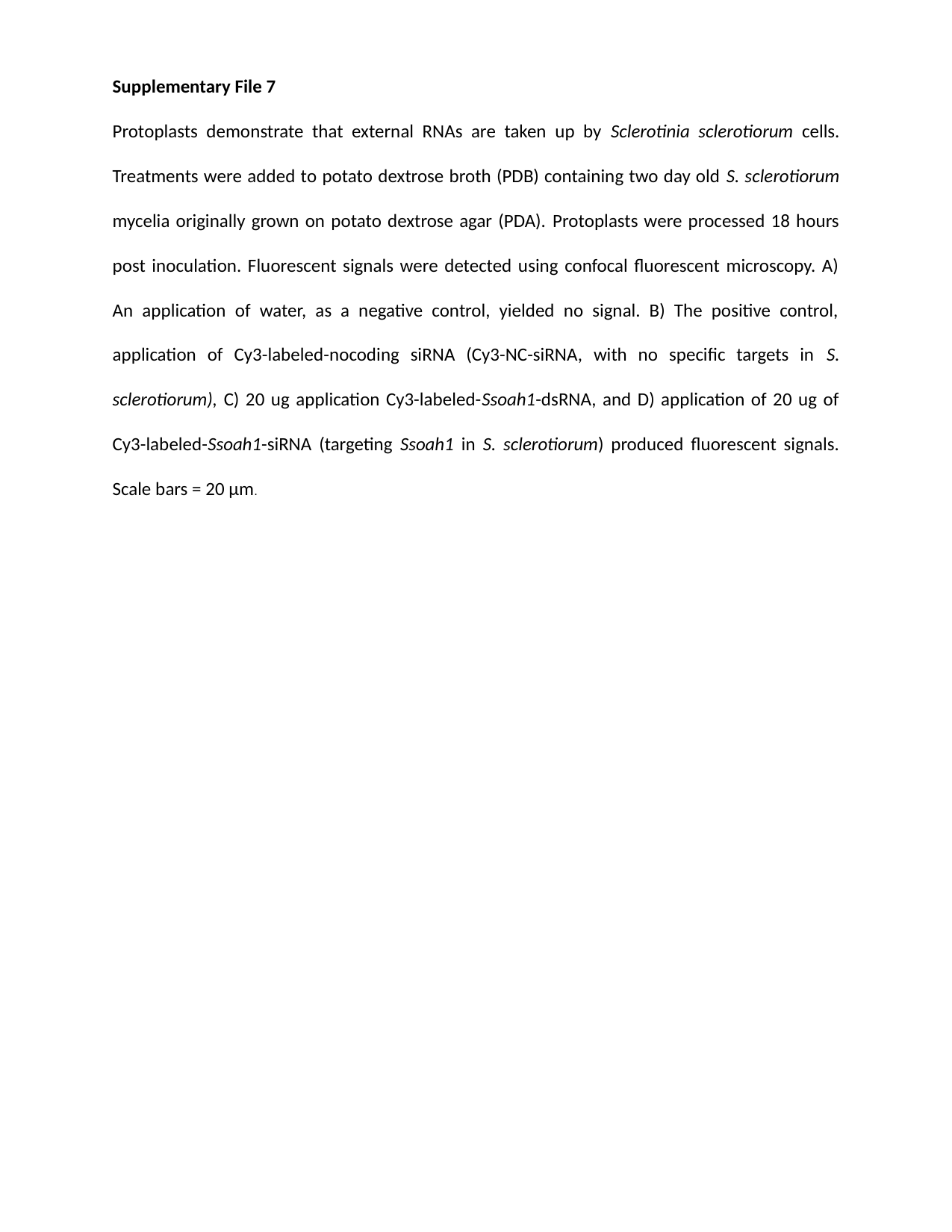

Supplementary File 7
Protoplasts demonstrate that external RNAs are taken up by Sclerotinia sclerotiorum cells. Treatments were added to potato dextrose broth (PDB) containing two day old S. sclerotiorum mycelia originally grown on potato dextrose agar (PDA). Protoplasts were processed 18 hours post inoculation. Fluorescent signals were detected using confocal fluorescent microscopy. A) An application of water, as a negative control, yielded no signal. B) The positive control, application of Cy3-labeled-nocoding siRNA (Cy3-NC-siRNA, with no specific targets in S. sclerotiorum), C) 20 ug application Cy3-labeled-Ssoah1-dsRNA, and D) application of 20 ug of Cy3-labeled-Ssoah1-siRNA (targeting Ssoah1 in S. sclerotiorum) produced fluorescent signals. Scale bars = 20 μm.

## Slide 9
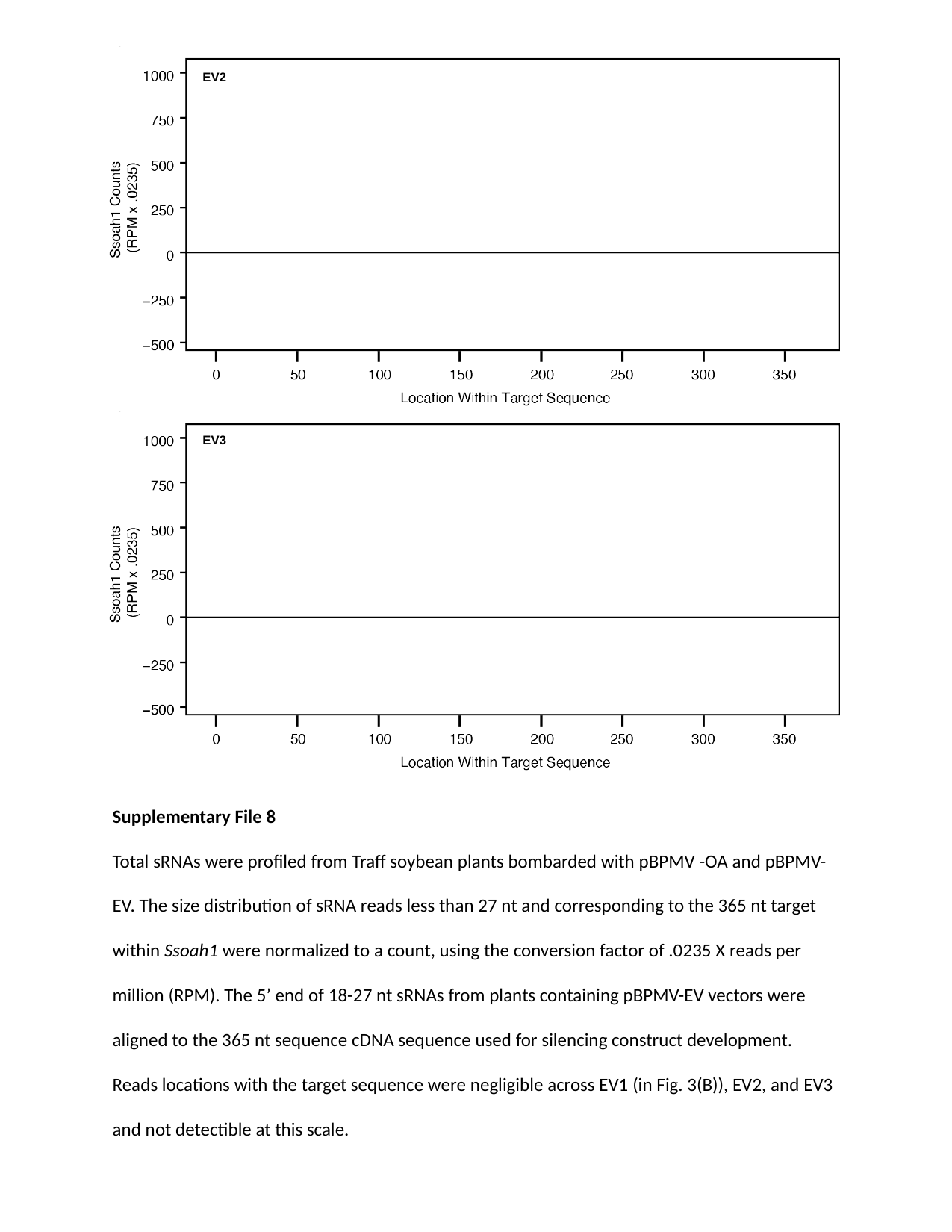

EV2
EV3
Supplementary File 8
Total sRNAs were profiled from Traff soybean plants bombarded with pBPMV -OA and pBPMV-EV. The size distribution of sRNA reads less than 27 nt and corresponding to the 365 nt target within Ssoah1 were normalized to a count, using the conversion factor of .0235 X reads per million (RPM). The 5’ end of 18-27 nt sRNAs from plants containing pBPMV-EV vectors were aligned to the 365 nt sequence cDNA sequence used for silencing construct development. Reads locations with the target sequence were negligible across EV1 (in Fig. 3(B)), EV2, and EV3 and not detectible at this scale.

## Slide 10
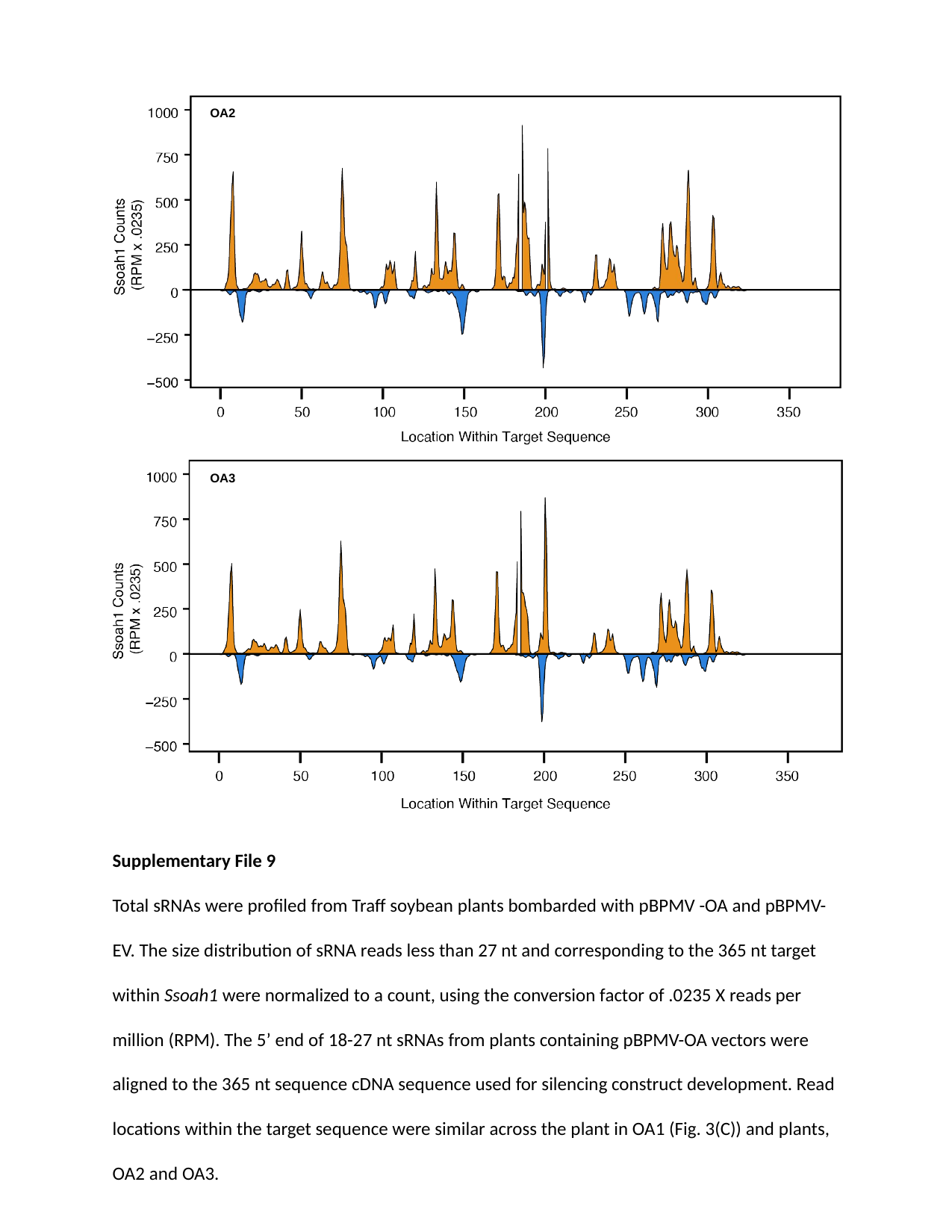

Supplementary File 9
Total sRNAs were profiled from Traff soybean plants bombarded with pBPMV -OA and pBPMV-EV. The size distribution of sRNA reads less than 27 nt and corresponding to the 365 nt target within Ssoah1 were normalized to a count, using the conversion factor of .0235 X reads per million (RPM). The 5’ end of 18-27 nt sRNAs from plants containing pBPMV-OA vectors were aligned to the 365 nt sequence cDNA sequence used for silencing construct development. Read locations within the target sequence were similar across the plant in OA1 (Fig. 3(C)) and plants, OA2 and OA3.
OA2
OA3
